# Intratympanic Steroid for the Management of Sudden Hearing Loss: Introduction of a Tapering Method

**DOI:** 10.22038/IJORL.2021.57477.2977

**Published:** 2022-01

**Authors:** Mohsen Rajati, Mohammad Mehdi Ghasemi, Mohammad-Reza Sharifian, Navid Nourizadeh, Razieh Yousefi, Masoumeh Hosseinpoor

**Affiliations:** 1 *Sinus and Surgical Endoscopic Research Center, School of Medicine, Mashhad University of Medical Sciences, Mashhad, Iran.*; 2 *Department of Biostatistics, Health School, Mashhad University of Medical Sciences, Mashhad, Iran.*

**Keywords:** Intratympanic, Steroid, Sudden sensorineural hearing loss

## Abstract

**Introduction:**

Sudden sensorineural hearing loss (SSNHL) is a therapeutic challenge. There are several controversies regarding the management protocol of SSNHL. This study aimed to present the results of a novel treatment algorithm, which is a combination of systemic steroids and a tapering intratympanic (IT) dexamethasone regimen.

**Materials and Methods:**

The past 10 years’ medical records of idiopathic SSNHL cases in Ghaem and Emamreza hospital, Mashhad University of Medical Sciences were evaluated. Patients were assessed using standardized methods for pure-tone threshold audiometry. The management method of SSNHL treatment included oral steroids combined with IT administration of dexamethasone once a day for 7 days and continuing it on an alternate day and then weekly basis. Patients’ recovery was assessed using Siegel’s criteria.

**Results:**

This study included a total of 248 cases of idiopathic sudden hearing loss, with a mean age of 40.63±16.19 years. In total, 105 (42.3%) and 143 (57.7%) patients were female and male, respectively. The most common associated symptoms included tinnitus (86.9%, n=205) followed by vertigo (52.1%, n=122). The final hearing outcome of patients showed that 39 (15.7%), 38(15.3%), 86 (34.7%), and 85 (34.3%) patients underwent a complete recovery, partial recovery, slight recovery, and no recovery, based on Siegel’s criteria.

**Conclusions:**

The dose, interval, and duration of IT steroid treatment were not universally approved. The treatment method designed based on a tapering of IT steroid injection in combination with already known systemic administration of steroids can be a treatment option in SSNHL patients.

## Introduction

Sudden sensorineural hearing loss (SSNHL) is described as an acute-onset hearing loss of 30 dB or greater over three or more frequencies within 72 h or less with no identifiable etiology (1). The annual incidence rate of SSNHL is estimated at about 10-20 cases per 100.000 people. However, the true rate seems to be higher due to many undiagnosed non-severe cases (2). Although the etiology of SSNHL is mostly unknown, a number of cases can be attributed to hemorheological disturbances, viral infections, immunological mechanisms, and even cardiovascular conditions (3, 4).

Some proposed treatment options for SSNHL include observation and close follow-up of patients (5), steroid therapy (6), acupuncture (7), hyperbaric oxygen therapy (8), and gunshot medications. However, the efficacy of early medical therapy and interventions is still controversial due to the inaccurate number of patients who recovered spontaneously from SSNHL, the delay in medical interventions in most patients, and several other confounding factors (9). 

Intratympanic (IT) steroid is recommended by the American Academy of Otolaryngology-Head and Neck Surgery when patients have incomplete recovery from SSNHL or after the failure of initial systemic management (10). The administration of IT steroids has been shown to result in higher inner ear concentrations than systemic steroids (5). The efficacy of IT injection of steroids has already been demonstrated in a retrospective longitudinal study, especially if performed within the first two weeks from the onset of hearing loss (9, 11). Simultaneous or sequential IT steroid administration is reported to be more effective than systemic steroids alone (12). No universally accepted protocol is available regarding the type of steroid, interval of injections, and duration of treatment (9). The present study reports the result of IT dexamethasone (IT-Dex) administration on a tapering base, combined with systemic oral steroid therapy. 

## Materials and Methods

The last 10 years (from January 2010 to January 2020) medical records of 460 patients diagnosed with SSNHL in the Otolaryngology Department of Ghaem University Hospital, Mashhad, Iran, were reviewed retrospectively in the present study. Afterward, the patients with mild hearing loss (pure tone audiometry threshold<40), diabetes, renal failure, history of any previous treatments for SSNHL, lack of follow-up, and a definite diagnosis of hearing loss, such as trauma or acoustic neuroma were excluded from the study.

All patients had unilateral SSNHL and were treated with both systemic (oral) and IT steroid therapy as first-line management. Informed written consent was obtained from all patients. The present study was approved by the Ethics Committee of Mashhad University of Medical Sciences, Mashhad, Iran (IR.MUMS.REC.1391.23).

The recorded data included gender, age, date of onset, accompanying symptoms, past medical history, background diseases, pure-tone audiogram, results of the paraclinical evaluations (i.e., lab tests and imaging), and prescribed medications. Hearing thresholds were measured at octave intervals between 250 Hz and 8 kHz. Audiograms were obtained before the beginning of treatment as well as two weeks and one month after treatment. 

***Audiometric data***

Patients were assessed using standardized methods for pure tone threshold audiometry. Pure tone average (PTA) was calculated as the mean of hearing thresholds at six frequencies (250, 500, 1,000, 2,000, 4,000, and 8,000 Hz), and Siegel’s criteria were used to assess the recovery (13, 14). Complete recovery was defined as a final hearing of more than 25 dB, while a partial recovery was defined as a hearing gain of more than 15 dB and a final hearing between 25 dB and 45 dB. A hearing gain of more than 15 dB and a final hearing poorer than 45 dB were accepted as a slight improvement. Moreover, a hearing gain of less than 15 dB and a final hearing poorer than 75 dB was considered no improvement as it was indicative of a non-serviceable ear.

***Management protocol***

The management method of SSNHL treatment includes the IT administration of dexamethasone (4 mg/ml) once a day for seven days. The injections would be stopped in case patients show either complete recovery or no improvement within the first week of treatment. However, in case of either partial recovery or slight improvement, the injections will continue on an alternate day regimen, and the audiogram will be repeated after one week. 

**Fig 1:**
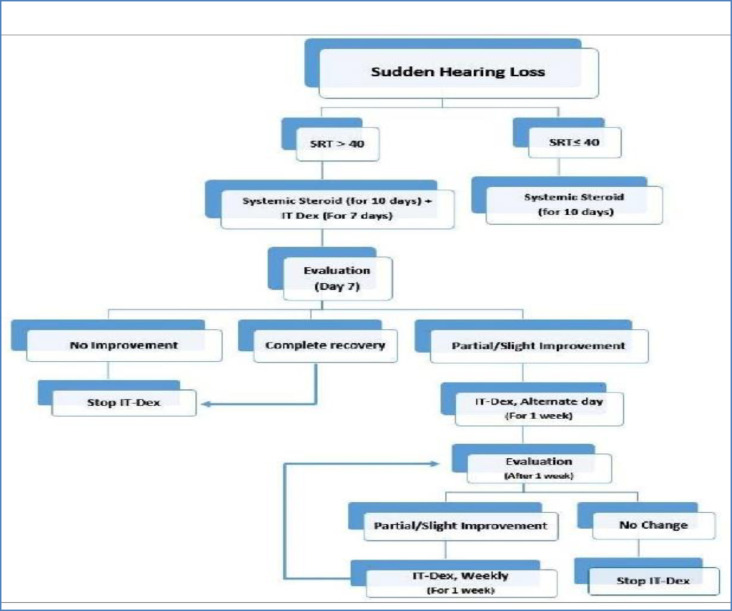
Algorithm for Mashhad intratympanic dexamethasone injection method

 Afterward, in case no improvement is observed, the IT injections will discontinue; however, the injections will continue on a weekly basis for two more weeks in case of any hearing improvement (Figure 1). 

Moreover, systemic therapy with oral prednisolone (1 mg/kg), ASA (80 mg), and acyclovir (400 mg/ five times a day) were used as the routine management of idiopathic SSNHL at our center. 

The IT injection procedure started with the evaluation of the integrity of the tympanic membrane with the aid of a microscope. Patients were placed in a supine position with their heads tilted 40-45° to the healthy side, and a 25-gauge spinal needle was introduced into the inferior part of the tympanic membrane. The middle ear was gently perfused with dexamethasone (4 mg/ml) and the patient was asked to avoid swallowing for five min. The patients were kept in this position for 15 min before discharge. 

***Statistical analysis***

Statistical analyses were performed in SPSS software (Version 16). The absolute numbers and percentages were computed to describe the data. Moreover, the $\chi^{2}$test and the independent t-test results were used to compare qualitative and quantitative variables, respectively. A p-value less than 0.05 was considered statistically significant.

## Results

In this study, a total of 248 cases of idiopathic sudden hearing loss were treated with daily IT administration of dexamethasone, according to the MITP. The mean±SD age of patients was 40.63±16.19 years, and 90 (37.2%) cases were within the age range of 40-60 years. Moreover, 105 (42.3%) and 143 (57.7%) cases were female and male, respectively. The most common associated symptoms were tinnitus (86.9%, n=205), followed by vertigo (52.1%, n=122). The hearing loss in patients was categorized as mild (26-40 dB), moderate (41-55 dB), moderately severe (56-70 dB), severe (71-90 dB), and profound (>90 dB), according to the mean pure tone threshold at 500, 1000, 2000, and 4000 Hz (13). 

Table 1 tabulates the frequency of pre-and post-treatment hearing levels. 

**Table 1:** Hearing loss classification at pre- and post-treatment

**Level**	**Pre-treatment**	**Post-treatment**
Mild (26-40dB)	Excluded	78 (31.5%)
Moderate (41-55 dB)	36 (14.5%)	38 (15.3%)
Moderately Severe (56-70 dB)	35 (14.1%)	36 (14.5%)
Severe (71-90 dB)	74 (29.9%)	59 (23.8%)
Profound (Above 90 dB)	103 (41.5%)	37 (14.9%)
Total	248(100.0%)	248(100.0%)

Table 2 tabulates the overall recovery outcomes during one month, according to Siegel’s criteria (13,14). 

**Table 2:** Overall recovery outcome

**Level**	**N**	**%**
CR	39	15.7
PR	38	15.3
SI	86	34.7
NI	85	34.3
Total	248	100.0


Subsequently, another analysis was made, and the cases were divided into two groups. 

Group 1 (good prognosis group) included patients with both complete or partial recovery and group 2 (poor prognosis group) included those with no or slight improvement. The two groups were compared in terms of several parameters presented in Table 3.

**Table 3:** Comparison of the two groups

Variable			Good Prognosis	Poor Prognosis	P-value
Age	<60	209	65 (31.1%)	144 (68.9%)	0.542
	≥60	33	8 (24.2%)	25 (75.8%)	
Gender	Male	143	46 (32.2%)	97 (67.8%)	0.412
	Female	105	31 (29.5%)	74 (70.5%)	
Smoking	No	174	57 (32.8%)	117 (67.2%)	0.650
	Yes	24	9 (37.5%)	15 (62.5%)	
Hypertension	No	173	49 (28.3%)	124 (71.7%)	0.180
	Yes	75	28 (37.3%)	47 (62.7%)	
Vertigo	No	112	48 (42.9%)	64 (57.1%)	0.000^*^
	Yes	122	26 (21.3%)	96 (78.7%)	
Tinnitus	No	30	13 (43.3%)	17 (56.7%)	0.210
	Yes	205	63 (30.7%)	142 (69.3%)	
Start of treatment from the onset (days)	<15	158	59 (37.3%)	99 (62.7%)	0.018^*^
	≥15	63	13 (20.6%)	50 (79.4%)	

Vertigo and the pretreatment hearing level were significantly different between the two groups, as indicated by the crosstab results. In addition, the time period before starting the treatment (within 15 days from the onset of hearing loss or after that) is another factor that significantly affects the outcome. 

## Discussion

Idiopathic SSNHL is a sudden onset hearing loss without a definite cause and is considered a medical emergency. Although the patients may occasionally recover spontaneously (15, 16), early treatment is recommended (12,17). The IT steroid therapy has gained wide popularity due to the theoretical advantage of an increased medication concentration at the target organ (inner ear) and the reduced systemic corticosteroid distribution and adverse effects (18,19). 

This study aimed to report the results of simultaneous first-line systemic steroids in combination with IT dexamethasone administered in a novel method. This method included daily injections followed by gradual tapering, based on the audiological status (Figure 1). Ermutlu et al. reported separate safe and effective steroids administration routes as a primary treatment for SSNHL (15). Kang et al. studied 494 patients and reported that IT steroid therapy is a safe alternative for systemic steroid therapy in the initial treatment of sudden hearing loss (12). According to Gianoli et al., IT steroids can be administered through a ventilation tube on four separate occasions over 10-14 days to patients who failed to respond to systemic treatment. Hearing improvement was observed in 10 out of 23 patients, presenting a 44% hearing salvage (20).

The IT administration method is still under debate. Several studies have used IT steroid injections to salvage the failure of systemic treatment. There are few studies on the simultaneous administration of IT and systemic steroids. Khorsandi Ashtiani reported that simultaneous administration of IT-Dex and oral prednisolone had no additional benefit (21). The IT administration intervals can be quite different. Kosyakov followed a 10-day protocol using a ventilation tube in a tapering approach (22), while Battaglia recommended the weekly usage for three weeks (23). Rauch also suggested four injections over two weeks (24), and Hong recommended daily injections for eight days (25). In the present study, daily doses were administered for one week with a gradual tapering to alternate days and then once a week (Figure 1). The degree of improvement cannot be easily compared in different studies regarding the application of various criteria. Siegle criteria were used in the present study, while in most studies PTA changes as little as 10 dB were regarded as hearing improvement (26). The PTA improved more than 15 dB in most cases (more than 65%) in the present study. Another important factor to be considered is that 41.5% of the included cases had profound HL, which was a poor prognostic factor. According to the collected data, the factors significantly related to hearing recovery in SSNHL included initial hearing threshold, vertigo, and the time of treatment initiation (being less than 15 days since the onset of hearing loss). Spontaneous hearing recovery, which tends to happen within two weeks from the onset, and the restoration of hearing during the early period of the disease can indicate the highest efficacy of IT steroid injection within 10 days after the onset (19). Tinnitus is the most frequent secondary symptom of SSNHL with a prevalence rate as high as 73-84% (24,27,28). Tinnitus is also known to disturb the patients more than hearing loss (29). 

Mühlmeier et al. reported that the high rate of tinnitus was associated with SSNHL at the onset and that there was a significant correlation between tinnitus and hearing loss in terms of recovery patterns and severity (30). According to the previous studies, around 76.2% and 61.9% of patients with SSNHL have such complications as tinnitus and vertigo, respectively (31,32). 

It has also been suggested that IT steroid is a good modality choice for the treatment of SSNHL patients who suffer from vertigo and tinnitus (33,34). In addition, it has been found that the IT steroid procedure resulted in a significant improvement of the symptom of tinnitus and vertigo (33, 35, 36).

## Conclusion

Currently, the IT steroid injection is a generally accepted method for the management of sudden hearing loss. Nevertheless, the dose, interval, and duration of the treatment are not universally approved. 

The treatment method can be rationally designed based on a tapering of IT steroid injection combined with already known systemic administration of steroids which seems to produce good results in these patients.
